# Supplementary material for: Enhancing Passion Fruit Resilience: The Role of Hariman in Mitigating Viral Damage and Boosting Productivity in Organic Farming Systems
Source: Int J Mol Sci. 2025 Feb 28;26(5):2177. doi: 10.3390/ijms26052177 (PMC11899903; doi:10.3390/ijms26052177)
Supplement: Supplementary file 1 [file ijms-26-02177-s001.zip › ijms-3429409-supplementary.pdf]

International Journal of
Molecular Sciences

MDPI

Supplementary Materials:

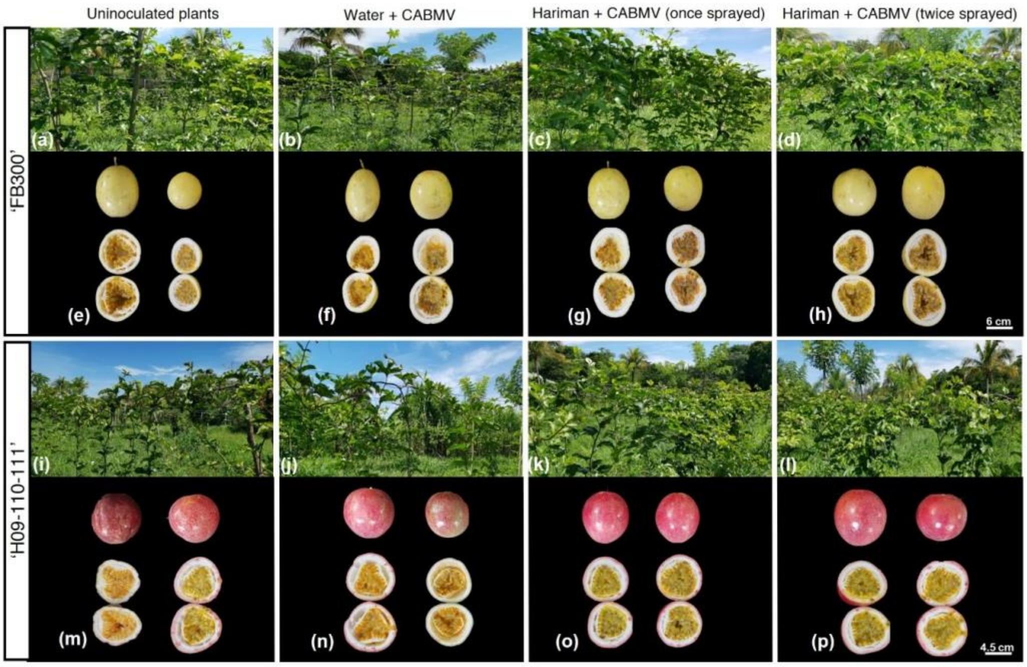

Figure S1. Development of whole passion fruit plants per treatment at 18 weeks after inoculation (wai) under field conditions, and overall appearance of the fruits. Panels (a-h) correspond to the 'FB300' genotype, while panels (i-p) correspond to the 'H09-110/111' genotype. CABMV infection resulted in reduced plant development in both uninoculated (natural infection) and water + CABMV treatments. The pulp of the fruits were bigger in plants treated with Hariman. The appearance of the fruit however was the same in once or twice spray.

Table S1. Hariman effect on the CABMV disease incidence (DI%) and disease severity (DS%) in passion fruit plants 'FB300' (a) and 'H09-110/111' (b) in greenhouse conditions. 2 at 9 weeks after CABMV inoculation (wai) are shown.

**(a) 'FB300'**	**2 wai**		**4 wai**		**6 wai**		**9 wai**	
**Treatments**	**DI%**	**DS%**	**DI%**	**DS%**	**DI%**	**DS%**	**DI%**	**DS%**
Uninoculated plants	0.0 ± 0.0b	0.0 ± 0.0b	0.0 ± 0.0b	0.0 ± 0.0b	0.0 ± 0.0c	0.0 ± 0.0b	0.0 ± 0.0b	0.0 ± 0.0b
Water + CABMV	66.6 ± 51.6a	52.0 ± 40.6a	100 ± 0.0a	81.6 ± 15.5a	100 ± 0.0a	83.7 ± 18.9a	100 ± 0.0a	95.0 ± 5.4a
Hariman + CABMV	0.0 ± 0.0b	0.0 ± 0.0b	16.6 ± 40.8b	13.7 ± 33.6b	50.0 ± 54.7b	31.2 ± 36.8ab	100 ± 0.0a	57.5 ± 20.7ab
**P value (*n*=6)**	*0.0017	**0.0082	*0.0001	**0.0008	*0.0003	**0.0022	*0.0001	**0.0003

**(b) 'H09-110/111'**								
**Treatments**	**DI%**	**DS%**	**DI%**	**DS%**	**DI%**	**DS%**	**DI%**	**DS%**
Uninoculated plants	0.0 ± 0.0c	0.0 ± 0.0b	0.0 ± 0.0c	0.0 ± 0.0b	0.0 ± 0.0b	0.0 ± 0.0b	0.0 ± 0.0b	0.0 ± 0.0b
Water + CABMV	100 ± 0.0a	86.1 ± 18.1a	100 ± 0.0a	98.6 ± 4.1a	100 ± 0.0a	98.6 ± 4.1a	100 ± 0.0a	98.6 ± 1.3a
Hariman + CABMV	55.5 ± 52.7b	37.5 ± 35.9b	55.5 ± 52.7b	56.1 ± 42.3b	77.7 ± 44.1a	70.8 ± 40.9a	88.9 ± 33.3a	66.6 ± 38.0b
**P value (*n*=9)**	*0.0001	**0.0001	*0.0001	**0.0001	*0.0001	**0.0001	*0.0003	**0.0001

Each value is the means (± standard deviation). Different letters in columns indicate significant differences between treatments.

*Corresponding results of One-Way ANOVA. Significant differences according to using the Bonferroni post-hoc test ($p < 0.05$).

**The P value is based on the Kruskal–Wallis test. Significant Dunn's multiple comparison test ($p < 0.05$).

Table S2. Effect of the use of Hariman and/or plant growth promoting bacteria (PGPB) in combination with the estimated productivity of passion fruit 'BGP418' from the EMBRAPA germplasm bank under field conditions. Total number of fruits (TNF), total yields (TY), weight fruit (WF) and pulp yield (PY).

Treatments	**TNF**	**TY (Tons ha^{-1})**	**WF (g)**	**PY (%)**
Control	21.5 ± 2.69 b	16.29 ± 0.29 c	341.0 ± 43.30 c	32.90 ± 0.78 a
Hariman (2x)	28.5 ± 1.11 a	22.10 ± 0.40 b	350.0 ± 7.90 bc	34.60 ± 1.45 a
PGPB (2x)	24.1 ± 1.31 ab	22.00 ± 0.27 b	410.0 ± 7.91 a	35.30 ± 1.13 a
Hariman + PGPB (2x)	30.1 ± 1.45 a	26.46 ± 0.61 a	396.0 ± 11.74 ab	32.40 ± 0.69 a
**P value***	0.0002	0.0001	0.0037	0.0224

Each value is the means (± standard deviation) of one experiment using 4 plants per treatment. Different letters in columns indicate significant differences between treatments according to using the Bonferroni post-hoc test ($p < 0.05$).

*Corresponding results of One-Way ANOVA.

Table S3. Effect of Hariman on relative CABMV accumulation in passion fruit plants under greenhouse and field conditions. Cowpea aphid-borne mosaic virus (CABMV) was detected in tissue samples of CABMV-inoculated P. edulis 'FB300' (a) and 'H09-110/111' (b) plants treated with water and Hariman by ELISA (PathoScreen® - Agdia) using standardized optical density (OD) values. The ELISA tests were conducted at 4, 8, and 12 weeks after inoculation (wai).

**a. 'FB300'**	**4 wai**			**8 wai**			**12 wai**			
	**Uninoculated**	**Water-treated + CABMV**	**Hariman-treated + CABMV**	**Uninoculated**	**Water-treated + CABMV**	**Hariman-treated + CABMV**	**Uninoculated**	**Water-treated + CABMV**	**Hariman-treated + CABMV**	**Hariman-treated x2 + CABMV**
	0.08	1.07	0.07	0.10	2.73	3.32	2.144	1.979	1.425	2.083
	0.07	1.70	0.06	0.11	2.58	3.45	1.895	2.32	1.709	1.861
	0.07	1.74	0.07	0.10	2.39	2.17	1.971	1.624	1.524	1.601
	0.07	1.52	0.07	0.09	2.44	2.72	1.488	2.376	1.588	1.355
	0.08	1.50	0.07	0.09	1.90	2.28	1.84	2.061	1.535	1.756
	0.07	1.36	0.08	0.09	2.01	2.12	1.896	1.867	1.57	1.698
	0.07	1.43	0.08	0.09	1.59	2.09	1.769	2.62	1.544	1.575
	0.07	2.20	0.14	0.10	2.54	2.01	1.37	1.992	1.43	1.514
	0.08	1.94	0.07	0.11	2.80	2.88	1.708	2.281	1.461	1.65
	0.07	2.23	0.08	0.09	2.66	3.16	1.414	2.337	1.531	1.618
	0.07	1.72	0.08	0.09	2.37	3.19	1.74	2.357	1.475	1.748
	0.07	1.67	0.08	0.10	2.46	3.24	1.363	1.744	1.379	1.293
**Average**	**0.07 b**	**1.67 a**	**0.08 b**	**0.10 b**	**2.37 a**	**2.72 a**	**1.72 b**	**2.13 a**	**1.51 b**	**1.65 b**
**SD**	**0.0039**	**0.3218**	**0.0177**	**0.0053**	**0.3469**	**0.5292**	**0.2448**	**0.2850**	**0.0846**	**0.2035**

**b. 'H09-110/111'**	**4 wai**			**8 wai**			**12 wai**			
	**Uninoculated**	**Water-treated + CABMV**	**Hariman-treated + CABMV**	**Uninoculated**	**Water-treated + CABMV**	**Hariman-treated + CABMV**	**Uninoculated**	**Water-treated + CABMV**	**Hariman-treated + CABMV**	**Hariman-treated x2 + CABMV**
	0.08	0.26	0.12	0.14	1.42	0.50	1.553	1.593	1.29	1.365
	0.07	0.28	0.12	0.11	1.27	0.42	1.312	1.473	1.188	1.262
	0.07	0.25	0.12	0.11	1.26	0.44	1.292	1.328	1.135	1.175
	0.07	0.40	0.11	0.11	1.16	0.39	1.284	1.126	1.05	1.242
	0.08	0.20	0.13	0.13	2.68	0.45	1.236	1.335	0.977	1.107
	0.07	0.10	0.15	0.10	2.73	0.46	1.25	1.387	1.082	1.031
	0.07	0.19	0.14	0.10	2.39	0.47	1.24	1.294	1.13	1.189
	0.07	0.12	0.12	0.11	2.33	0.51	1.155	1.326	1.144	1.112
	0.08	0.19	0.09	0.10	1.86	0.66	1.252	1.324	1.09	1.072
	0.07	0.10	0.08	0.09	1.86	0.51	1.245	1.285	0.909	1.167
	0.07	0.18	0.13	0.09	3.34	0.35	1.245	1.374	1.084	1.011
	0.07	0.18	0.12	0.09	3.15	0.43	1.172	1.247	1.082	1.132
**Average**	**0.07 a**	**0.20 a**	**0.12 a**	**0.11 b**	**2.12 a**	**0.46 b**	**1.27 ac**	**1.34 a**	**1.10 b**	**1.16 bc**
**SD**	**0.0039**	**0.0815**	**0.0175**	**0.0135**	**0.7276**	**0.0748**	**0.0955**	**0.1100**	**0.0926**	**0.0968**

Different letters in columns of average indicate significant differences between treatments.

The statical analysis is based on non-parametric the Kruskal–Wallis test. Significant Dunn's multiple comparison test ($p < 0.05$).
